# Strong Coupling to Circularly Polarized Photons: Toward Cavity-Induced Enantioselectivity

**DOI:** 10.1021/acs.jpclett.4c01701

**Published:** 2024-08-21

**Authors:** Rosario R. Riso, Enrico Ronca, Henrik Koch

**Affiliations:** † Department of Chemistry, Norwegian University of Science and Technology, 7491 Trondheim, Norway; ‡ Department of Chemistry, Biology and Biotechnology, University of Perugia, Via Elce di Sotto 8, 06123 Perugia, Italy; § Scuola Normale Superiore, Piazza dei Cavalieri 7, 56126 Pisa, Italy

## Abstract

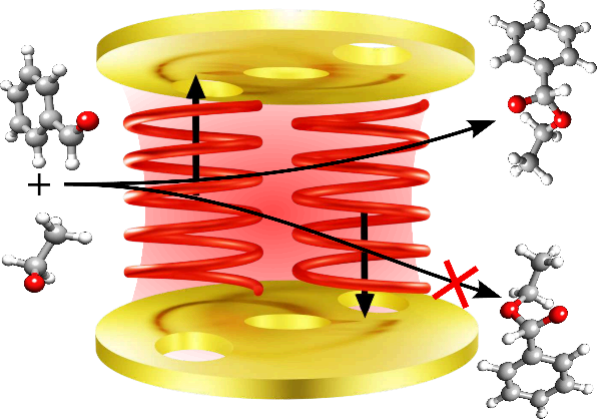

The development of new methodologies for the selective synthesis of individual enantiomers is still one of the major challenges in synthetic chemistry. Many biomolecules, and also many pharmaceutical compounds, are indeed chiral. While the use of chiral reactants or catalysts has led to substantial progress in the field of asymmetric synthesis, a systematic approach applicable to general reactions has still not been proposed. In this work, we demonstrate that strong coupling to circularly polarized fields can induce asymmetry in otherwise nonselective reactions. Specifically, we show that the field induces stereoselectivity in the early stages of chemical reactions by selecting an energetically preferred direction of approach for the reagents. Although the effects observed thus far are too small to significantly drive asymmetric synthesis, our results provide a proof of principle for field-induced stereoselective mechanisms. These findings lay the groundwork for future research.

Asymmetric synthesis is a formidable challenge in chemistry, as demonstrated by the 2021 Nobel Prizes to professors Benjamin List and David MacMillan.^1−5^ Most biologically relevant molecules belong to a special class of systems that cannot be superimposed with their own mirror image. These molecules, known as chiral molecules, exist in two possible configurations called enantiomers, which have identical energy levels and share most physical properties. As a result, achieving a selective synthesis of only one of the two enantiomers is a complicated task and reactions often produce a mixture of enantiomers instead of an enantiomerically pure product. This is a major complication in the pharmaceutical industry since most often only one mirror image has the desired healing behavior, while the other may be ineffective or even harmful.^6^ Living organisms have developed remarkable techniques to produce only one of the two enantiomers through the use of chiral enzymes and catalysts.^7,8^ As a result, the most successful research lines in asymmetric synthesis have also mostly focused on the development of new and more efficient catalysts.^4,9^ However, catalysts are often reaction specific and a systematic approach is yet to be developed.^5^ Strong coupling between molecules and vacuum photons fields has recently emerged as a promising technique to engineer molecular properties in a noninvasive way.^10−14^ When light and matter interact strongly, for example inside optical cavities, they form new hybrid electron-photon states with unique properties: the polaritons.^15−17^ Intriguing applications of polaritonic chemistry include modifying molecular absorption and fluorescence,^18−20^ altering photochemical processes^21^ and even speeding up or slowing down chemical reactions.^22−25^ In a previous work, we demonstrated that circularly polarized fields can induce energy differences between enantiomers, even in the ground state. This result stems from the fact that the two enantiomers exhibit differential absorption with respect to left- and right-circularly polarized light (LHCP and RHCP), a phenomenon known as circular dichroism. Notably, these energy differences can be exploited to create enantioselective signatures in the rotational spectra of the two mirror images.^26^

In this work, we investigate whether strong coupling to circularly polarized fields has the potential to induce enantioselectivity in reactions that are normally nonselective. Indeed, while the effect on the formed enantiomer is intriguing on itself, an even more captivating prospect is whether the field can bias nonchiral reagents toward favoring the formation of one configuration over the other (see Figure 1a). In particular, we suggest that appropriate engineering of the field can lead to an imbalance in the approach direction of the reagents, leading to the preferential formation of one of the two possible products.^27−29^ Furthermore, since strong coupling to quantized fields induces long-range correlation between molecules, we show that the footprint of the field becomes increasingly relevant at large distances where the electronic effects become smaller and smaller. Our studies suggest that by harnessing the power of quantum fields, it is possible to induce enantioselectivity in chemical processes. However, further investigations are needed as the effects reported in this work are not intense enough to alter reactions at room temperature.

**Figure 1:**
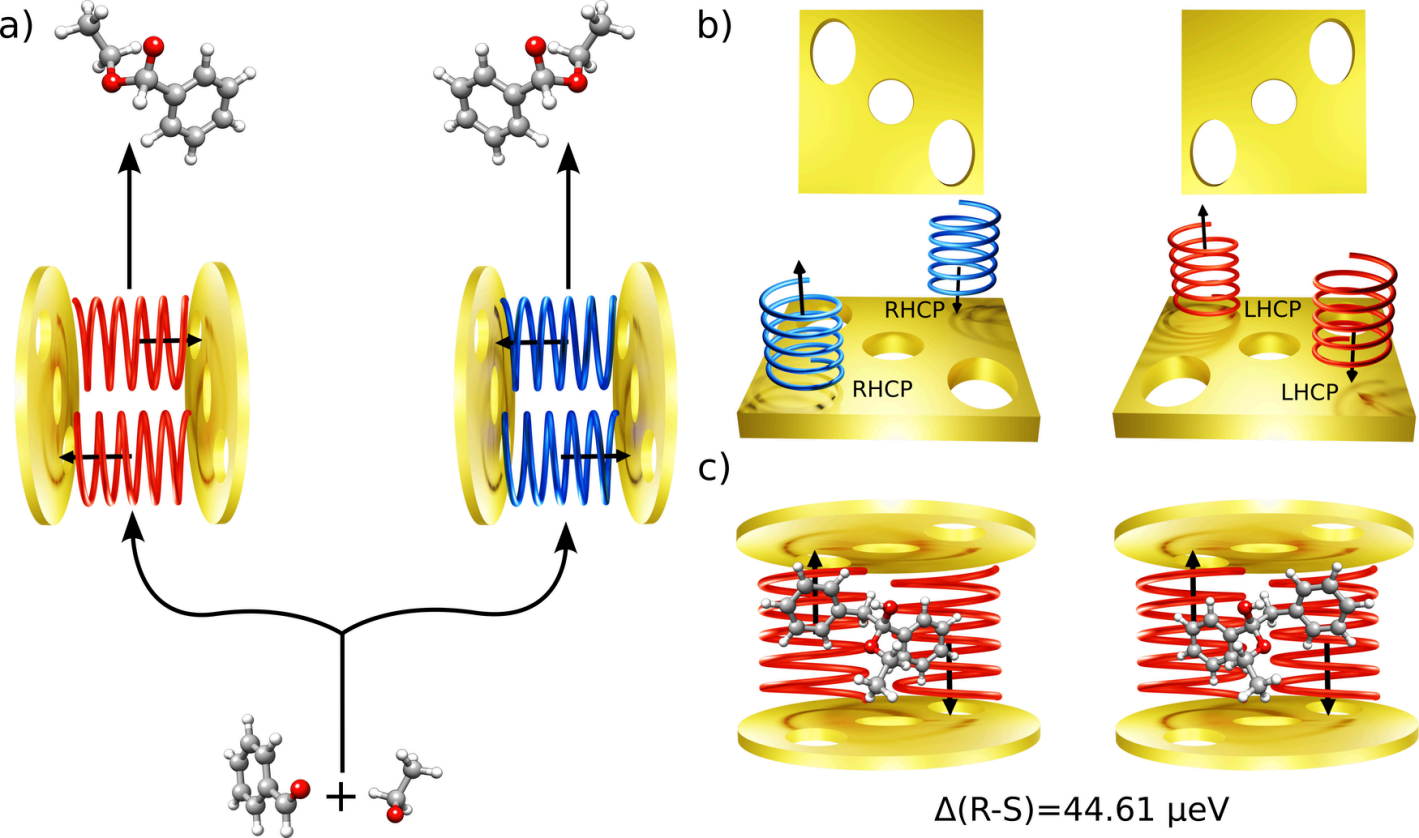
**Chiral cavities and their discriminating power.** (a) Pictorial representation of a benzaldehyde reacting with ethanol inside an LHCP (red on the left) and RHCP (blue on the right) chiral cavity. The main idea of this paper is that strong coupling to circularly polarized fields might induce a bias toward the formation of a preferred enantiomer. (b) Example of mirrors reflecting only RHCP and LHCP light without changing the field circular polarization, as reported by Baranov and co-workers.^30,31^ (c) Ground state energy difference between two enantiomers of 1-ethoxy-1,2-diphenylethanol in an LHCP cavity.

Strong coupling between molecules and circularly polarized light can be achieved using chiral cavities, devices that confine only one circular polarization of the field within a reduced volume, known as the quantization volume.^32−36^ Experimental realization of such devices has been reported by Gautier et al. and Wu et al.^37,38^ and has been theorized by Baranov and co-workers,^30,31^ see Figure 1b for a pictorial representation. Exploiting the unique properties of the circularly polarized fields, chiral cavities effectively break the energy degeneracy between the two mirror images of a chiral molecule. To illustrate this phenomenon, we report in Figure 1c the field-induced energy differences between the two enantiomers of a chiral molecule, 1-ethoxy-1,2-diphenylethanol, placed inside an LHCP chiral cavity. The calculation is performed at the minimal coupling quantum electrodynamics coupled cluster level (see Computational Details in the Supporting Information (SI)).^26^ The field frequency is set at ω = 2.7 eV and the light-matter coupling, λ, is set at 0.05 atomic units (a.u.). The coupling strength is a critical parameter in determining the magnitude of the polaritonic effects. In particular, when λ = 0 au, photons and matter are decoupled and no cavity effects are observed. An increase in λ, instead, signals that light and matter interact more intensely leading to larger polaritonic effects. Large λ values are achieved by confining the field quantization volume, *V*, as . An increase in λ, therefore, comes at the expense of the space that is affected by the field. In line with ref (26), we refer to the enantiomeric energy differences induced by the cavity as the field discriminating power. For the 1-ethoxy-1,2-diphenylethanol molecule in Figure 1c, the ground state field discriminating power is on the order of 40 μeV, consistent with the findings reported previously in the literature.^26,39^ Even though this energy range can be experimentally detected using modern instruments (e.g., nuclear magnetic resonance operates in a similar energy range), it is important to note that the chiral effects are several orders of magnitude smaller than both room temperature thermal energy and molecular binding energies, respectively on the order of 25 meV and hundreds of meV.^40,41^ Although the field-induced discrimination increases for larger chiral systems and stronger light-matter coupling strengths, the magnitude of the discriminating power remains within the μeV range per molecule for real systems.

In refs (26) and (42), we show that since the cavity field introduces a spatial anisotropy, the molecular energy is highly dependent on the system orientation. As a consequence, during a reactive event every reaction pathway is either stabilized or destabilized by the field.^43,44^ This suggests that circularly polarized fields can influence the stereoselectivity of chemical reactions by favoring specific approach directions. To investigate this idea, we focus on the field effects on the nonenantioselective reaction between benzaldehyde and ethanol leading to the formation of a hemiacetal (see Figure 2a). The approach direction of the reagent plays a crucial role in determining the chirality of the final product. For the benzaldehyde and ethanol reaction, the orientation of the alcohol group relative to the aromatic ring is the key variable. A crossing of this plane results in a chirality inversion of the product. In Figure 2 we illustrate two different approach directions of the alcohol group: from left and right with respect to the aromatic ring plane. In particular, the benzaldehyde and the ethanol approach each other parallel with respect to the field wave vector **k**. Similar results are obtained by studying the case where one reagent approaches the other perpendicularly to **k**, as shown in SI Figure 3, or by changing the field frequency, as shown in SI Figure 4. The geometries in Figure 2 are allowed to relax along the reaction pathway such that the only constrained degree of freedom is the distance between the ethanol oxygen and the aldehyde carbon. The chiral cavity utilized in these calculations is an RHCP cavity with an optical frequency of ω = 2.7 eV and a coupling strength of λ = 0.05 au. In Figure 2b, we show the potential energy surface (PES) of the reaction while in Figure 2c we report the energy difference between the two approach directions, (left minus right), as a function of the distance. We note that in the noncavity case, the energy difference in Figure 2c is zero because the two paths are mirror images of one another and the electronic Hamiltonian contains no parity-violating terms. The chiral field can instead discriminate between the two approach directions leading to a net difference in the PES profile. The field-induced energy difference between reactive pathways leading to two different enantiomers is exactly the effect this work focuses on. The sign of the field stabilization is constant along the reactive path, indicating that the cavity consistently favors the right approach of the alcohol in Figure 2a, favoring an *R* enantiomer product. Nonetheless, the electronic effects overwhelmingly dominate compared to the contributions from the cavity, which remain in the μeV range, and only a negligible fraction of the reactive pathways will be redirected due to the effects discussed in Figure 2. However, we notice that the cavity stabilization of the approach creating an *R* enantiomer increases as the distance between the reagents grows. This is a crucial observation because while the electronic effects diminish quite rapidly with distance, strong coupling to quantized fields introduces long-range correlation between molecules.^28^ In an ideal gas phase experiment, where the reagents are approaching from a large distance, the discriminating power of chiral fields might therefore become the dominating effect.

**Figure 2:**
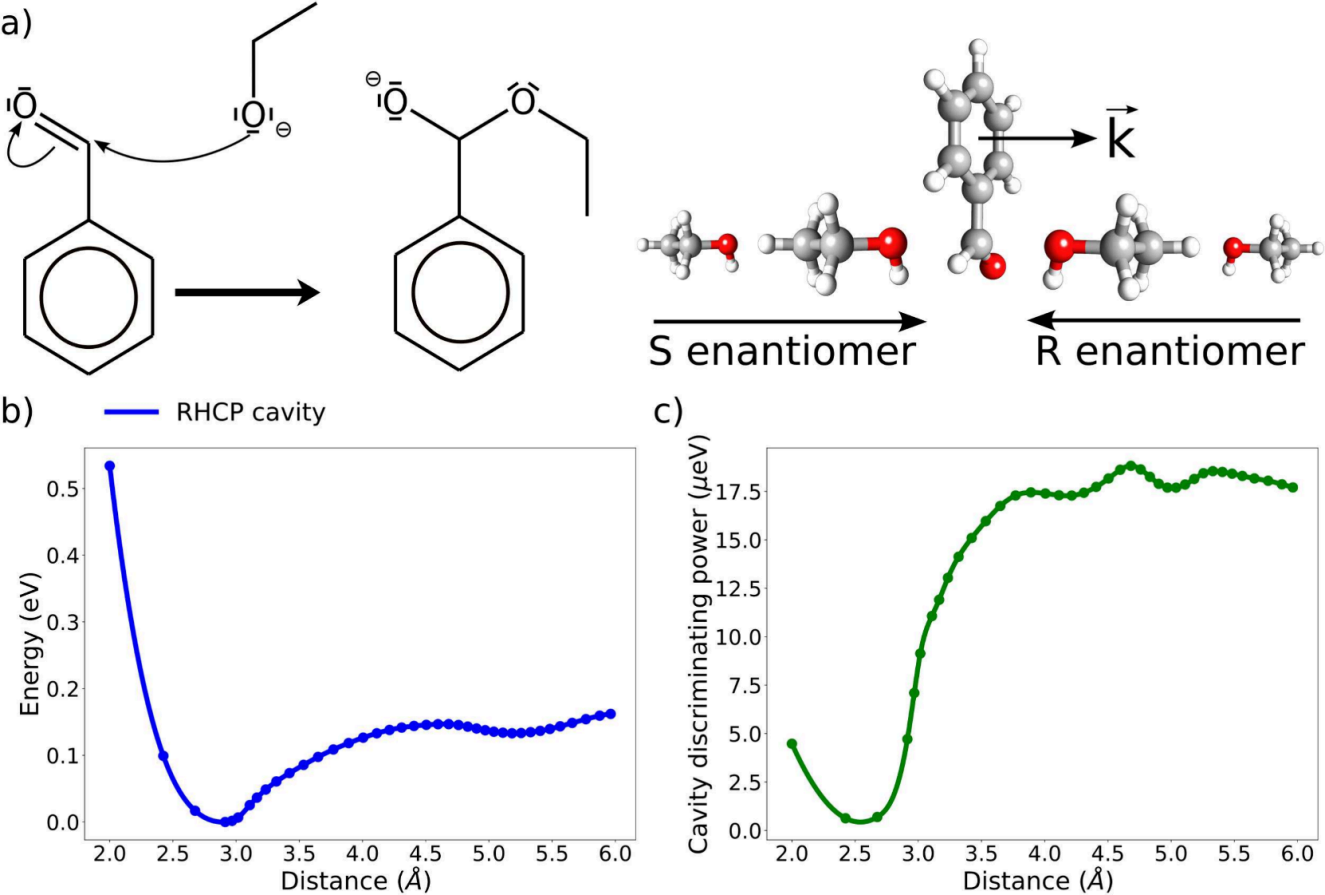
**Short range field effects on the benzaldehyde–ethanol reaction.** (a) Reaction mechanism for the benzaldehyde–ethanol reaction. The reaction is nonenantioselective. (b) Potential energy surface for the reaction as a function of the C–O distance. The ethanol is approaching parallel to the cavity wave vector **k**. (c) Cavity discriminating power, computed by subtracting the potential energy surfaces for *S* and *R* approaches (see (a)). We notice that the sign of the field discrimination power remains constant along the full pathway.

To exemplify the persistence of the cavity discriminating power for large separations, we illustrate the field effects for the benzaldehyde-ethanol reaction when the reagents are 200 Å apart. Specifically, Figure 3 displays the angular dispersion of the energy for an RHCP and an LHCP cavity, with the field-induced effects computed as the difference between the red and the blue curves. The discriminating power of the cavity (obtained by subtracting the LHCP results from the RHCP results) exhibits a prominent sign change around the 90° mark, i.e. where the system’s chirality is inverted. Specifically, RHCP fields stabilize the *R* enantiomer (θ less than 90°) more than the *S* enantiomer (θ larger than 90°), while the opposite behavior is observed in the LHCP cavity. This result validates the intuitive picture that when the system inverts its chirality, the field stabilization also changes sign. Although the cavity-induced discrimination remains in the μeV range, it is crucial to consider that in Figure 3, the Coulomb interactions between the reagents are negligible and the field effects can accumulate over the whole reaction path. The shape of the angular dispersion is predominantly determined by dipole effects, which are larger than the enantiomeric discrimination. However, at these large distances, the enantioselective field effects are comparable to the dipole effects and the dispersion curves for the rotation in the RHCP and LHCP cavities are therefore qualitatively different.

**Figure 3:**
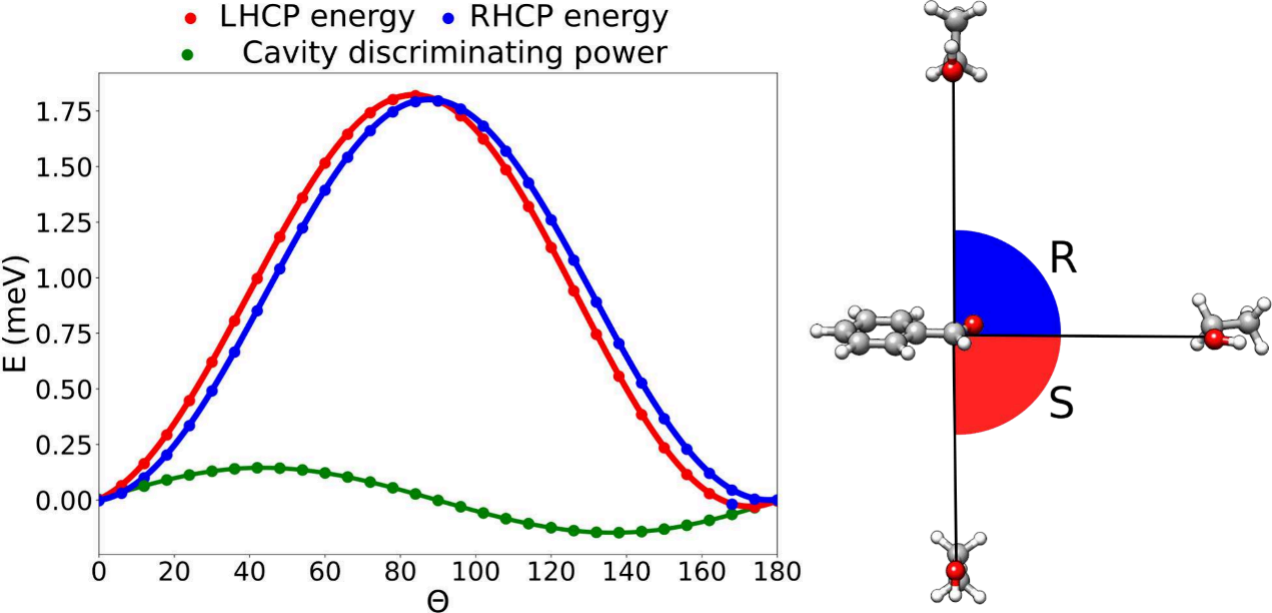
**Angular dispersion of the energy for the hemiacetal reaction with the reagents placed 200 Å apart.** The sign change in the chiral discriminating power shows the inversion of the preferred chirality between LHCP and RHCP cavities.

At long distances, it is critical to account for orientational effects as the molecules are free to reorient with respect to each other. To determine whether the orientational effects will cancel the long-range contributions of the cavity discriminating power, we present in Figure 4 the field-induced energy differences between the top and bottom reactions for all possible approach directions of the alcohol in an RHCP cavity, as obtained by varying the spherical angles θ and ϕ. The plot in Figure 4 clearly presents peaks and valleys, with the deepest minimum (in blue) corresponding to the path where the *R* enantiomer formation is maximally favored, and the highest maximum (in red) indicating the approach direction that most significantly favors the *S* enantiomer. Furthermore, we notice that the minima are more pronounced than the maxima.

**Figure 4:**
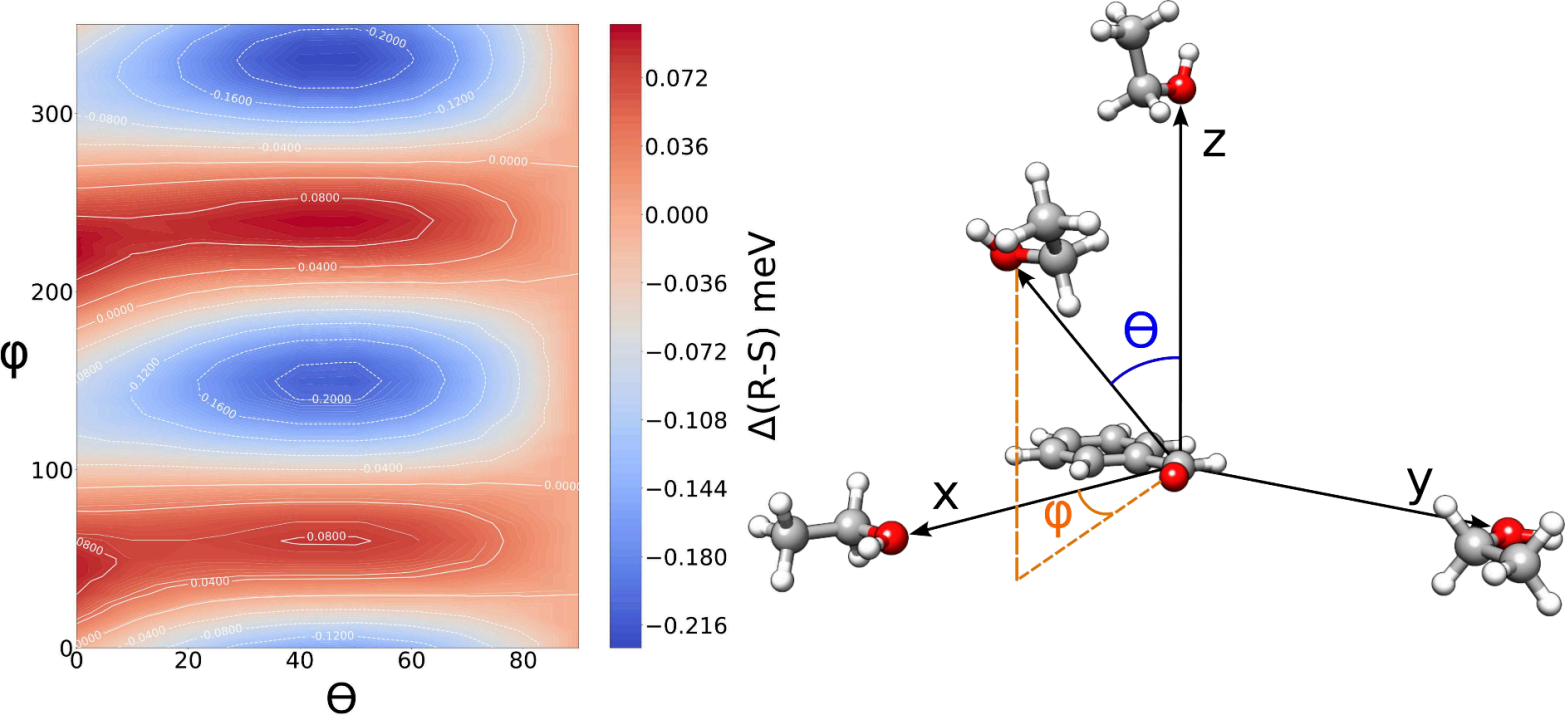
Field-induced stabilization of the *R* (blue) and *S* (red) enantiomers as a function of the relative orientation between the two reagents.

When a reaction is carried out inside a chiral cavity, like in Figure 4, every configuration of the potential energy surface is populated following the Boltzmann distribution:
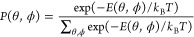
1where *P*(θ, ϕ) is the probability of finding the molecule in a certain configuration, *T* is the reaction temperature, and *k*_B_ is the Boltzmann constant. In Figure 5a, we compare the probability of an approach from above (blue hemisphere) with an approach from below (red hemisphere) for the benzaldehyde–ethanol reaction at different distances. To check the robustness of the results over the reaction parameters, we consider the reactive approach at different temperatures: 298 K (room temperature), 273 K (0 °C), 183 K, and 77 K (nitrogen condensation). The results in Figure 5a demonstrate that the field-induced effects persist even after rotational averaging. In an RHCP cavity, strong coupling to the field creates a small bias toward the formation of the *R* enantiomer. This outcome could have been predicted by analyzing the orientational surface displayed in Figure 4, where the *R* enantiomer is on average more stabilized by the field. The same enantiomer is favored at all distances and for all temperatures confirming that the cavity discriminating power is robust. Furthermore, the field discrimination is more pronounced at larger distances. This is a crucial observation because, as the Coulomb interaction becomes negligible, the chirality effects dominate the interaction between the fragments. Specifically, in Figure 5b we plot the magnitude of the Coulomb and chiral interactions for the approach at θ = 40° and ϕ = 20° (see Computational Details). We observe that the field-induced discrimination is significantly larger than the Coulomb contribution for distances larger than 50 Å. As expected, the cavity effect on the stereoselectivity increases as the temperature decreases because the lower energy approach direction is more populated at lower temperatures. The field-induced enantioselectivity reported in Figure 5 may be small, with only a few fractions of a percentage gained even at 77 K. However, it is important to note that the effect is not zero even at very large distances and that the chirality effects at all distances would therefore accumulate to influence the reaction outcome. We notice that for the 77 K case, the discrimination at a 50 Å distance is still quite intense compared to the 200 Å discrimination. This is because at low temperatures very few approach directions are thermally available and the chirality effects on those few approach directions dominate the discrimination, effectively limiting the averaging. The results in Figures 3 and 5 are intended as purely theoretical since it would not be possible to experimentally use these long-range effects while maintaining the light-matter coupling strength at 0.05 au. In that setup, indeed, maximum distances could realistically reach 20 Å. For experimental setups where the distances reported in Figure 5 can be realized, the coupling strength would be equal to λ = 0.005 au. Since the ground state effects display a λ^2^ dependence,^45^ the field discrimination would also decrease by a factor 100. However, the qualitative mechanisms of chiral discrimination do not change if λ is decreased and the same effects would come into play in experimental setups. Our results establish the conceptual framework for understanding the potential impact of strong coupling to circularly polarized fields on asymmetric synthesis. However, careful engineering of the field and reaction conditions would be needed to observe a significant impact on the chirality of the final product.

**Figure 5:**
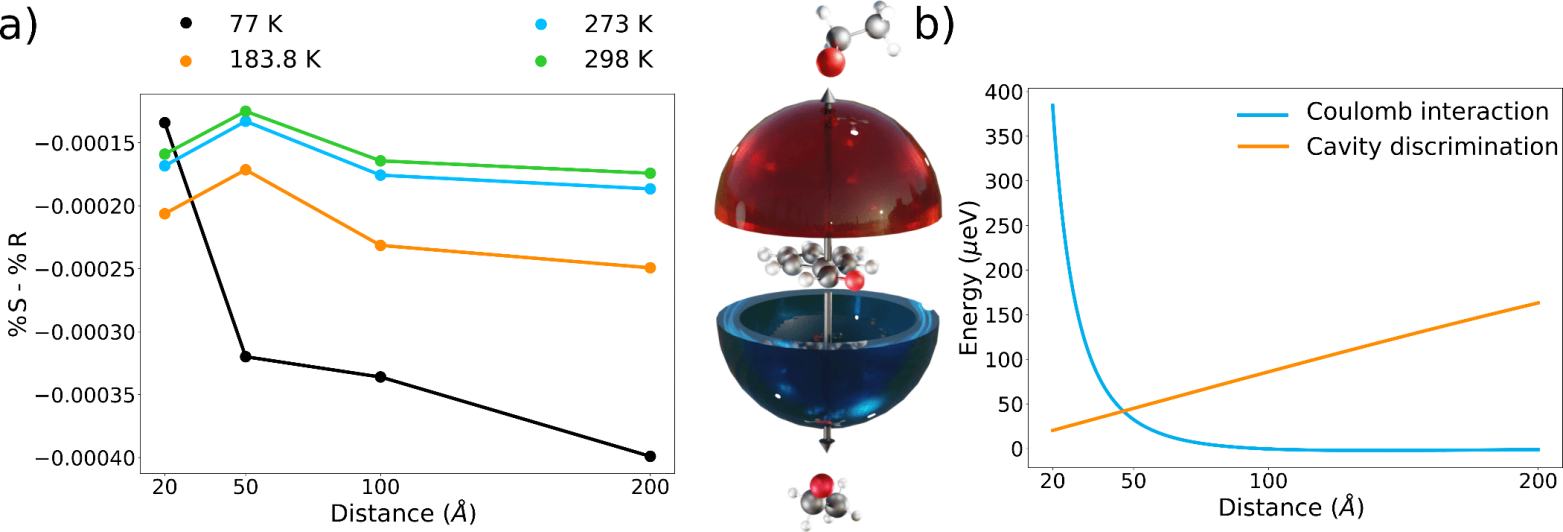
(a) Field-induced enantioselectivity as a function of the distance and the reaction temperature. We notice that the enantioselectivity always displays the same sign, becoming more pronounced for larger distances. (b) The Coulomb interaction between the fragments decays much faster than the field-induced effects.

In ref (26), we demonstrate that the field-induced discrimination of a chiral cavity is directly linked to the optical activity of the molecule. This observation substantiates the claim that field-induced energy discriminations arise because of circular dichroism. This is a remarkable result since it connects to the idea of homochirality, where the field promotes the enantiomer with the same chirality.^33^ A more detailed investigation of the homochirality hypothesis in chiral cavities will be the subject of future investigations as our findings indicate that the connection between optical rotatory power and field stabilization is not straightforward (only the positive pole of the optical activity determines the effect). Even so, our results show that for every reaction a field polarization can be chosen to favor a desired enantiomer. The field discriminating effects can also be attributed to a different mechanism, well established in the chemistry community. When dealing with a racemic mixture containing both enantiomers, one effective approach to separate the two involves the reaction of the mixture with an enantiomerically pure system. Despite still being isomers, the two reaction products are indeed no longer mirror images of one another; they are instead diastereoisomers. Diastereoisomers have different physical and chemical properties and therefore techniques such as distillation, crystallization, chromatography, or extraction can be employed to separate them.^46^ Nowadays this procedure is not used as much because the reactive steps significantly reduce the yield of the separation process. The field discriminating power in chiral cavities is similar to the diastereomeric mechanism described above. Specifically, while the two enantiomers of a chiral molecule have the same energy outside the chiral cavities, upon interaction with a chirally pure entity, the circularly polarized field in our framework, two different diastereoisomers are formed. Inside a chiral cavity, no symmetry operation can interconvert one enantiomer into the other.^26^ While drawing an analogy between light-matter interaction and chemical bonding might appear bold, in the strong coupling regime the field effects often mirror molecular interactions, see the solvent caging effect reported by Li et al.^20,47^

Regardless of the conceptual framework chosen to rationalize the discriminating power of the field, our work demonstrates that strong coupling between molecules and tightly confined circularly polarized fields leads to enantioselective effects even in nonselective reactions. Despite being small compared to the electronic effects, the cavity discriminating power becomes increasingly relevant at large separations. The field does indeed create long-range correlation between the fragments that can reorient at long distances. Overall, this research shines a new light on how strong coupling to circularly polarized fields may open new and unexplored paths toward asymmetrical synthesis. Considering the recent fast advances made from the experimental side in the fabrication of chiral cavities,^30,31,37,38,48^ we believe that the proposed ideas will, in the near future, find an experimental use. Future theoretical efforts will be devoted to studying the field-induced discrimination for excited states with a particular focus on collective effects.^32,33,49^ Additionally, the study of strong coupling to circularly polarized fields in the vibrational energy^50^ range will be the subject of further investigations.

## Data Availability

The geometries used for the reported calculations can be found on the Zenodo open repository.^51^ The data that support the findings of this study are available at the Zenodo link.^51^ The *e*^*T*^ program^52^ is open-source, and installation instructions can be found at the Zenodo link.^51^

## Supporting Information Available

Additional data on computational details, an S_N_1 reaction case, and the cavity effects on van der Waals complexes (PDF)
