# Supplementary material for: Strong Coupling to Circularly Polarized Photons: Toward Cavity-Induced Enantioselectivity
Source: J Phys Chem Lett. 2024 Aug 21;15(34):8838–44. doi: 10.1021/acs.jpclett.4c01701 (PMC11372830; doi:10.1021/acs.jpclett.4c01701)
Supplement: Supplementary file 1 — jz4c01701_si_001.pdf [file jz4c01701_si_001.pdf]

Supporting Information to: Strong Coupling to Circularly Polarized Photons: towards cavity-induced enantioselectivity

Rosario R. Riso,[†] Enrico Ronca,[*,‡] and Henrik Koch[*,†,¶]

[†]*Department of Chemistry, Norwegian University of Science and Technology, 7491 Trondheim, Norway*

[‡]*Department of Chemistry, Biology and Biotechnology, University of Perugia, Via Elce di Sotto, 8, 06123, Perugia, Italy*

[¶]*Scuola Normale Superiore, Piazza dei Cavalieri 7, 56126 Pisa, Italy*

E-mail: enrico.ronca@unipg.it; henrik.koch@ntnu.no

Computational details

The interaction between the electrons and the field is modeled using the Born-Oppenheimer minimal coupling Hamiltonian

$$\begin{aligned} H =& \frac{1}{2}\sum_i \left(\mathbf{p}_i - \mathbf{A}(\mathbf{r}_i)\right)^2 + \sum_{i>j}\frac{1}{|r_i - r_j|} + \sum_{I>J}\frac{Z_I Z_J}{|R_I - R_J|} - \sum_{i,I}\frac{Z_I}{|R_I - r_i|} \\ &+ \frac{1}{2}\int \left(\mathbf{E}^2(\mathbf{r}) + c^2\mathbf{B}^2(\mathbf{r})\right) d^3r, \end{aligned} \tag{1}$$

where i and j label electrons while I and J label nuclei with charges Z_I and Z_J. The vector potential, the electric and the magnetic fields are denoted by $\mathbf{A}(\mathbf{r})$, $\mathbf{E}(\mathbf{r})$ and $\mathbf{B}(\mathbf{r})$, respectively. In the strong light-matter coupling regime, the field is a critical component of the system and therefore the quantum nature of the photons needs to be taken into account. This is achieved by adopting a quantum electrodynamics (QED) description. Inside a chiral cavity, the field is circularly polarized, i.e. the second quantized vector potential is given by[1–3]

$$\mathbf{A}_{\pm}(\mathbf{r}) = \sum_{\mathbf{k}} \frac{\lambda}{\sqrt{2\omega_k}}\left(\boldsymbol{\epsilon}_{\mathbf{k}\pm} e^{i\mathbf{kr}}\left(b_{\mathbf{k}} + b^{\dagger}_{-\mathbf{k}}\right) + \boldsymbol{\epsilon}^*_{\mathbf{k}\pm} e^{-i\mathbf{kr}}\left(b^{\dagger}_{\mathbf{k}} + b_{-\mathbf{k}}\right)\right), \tag{2}$$

where $b_{\mathbf{k}}/b_{-\mathbf{k}}$ and $b^{\dagger}_{\mathbf{k}}/b^{\dagger}_{-\mathbf{k}}$ respectively create and annihilate a photon with wave vector $\pm\mathbf{k}$ and polarization $\boldsymbol{\epsilon}_{\mathbf{k}\pm}$. The $\pm$ subscript in Eq.2 refers to the circular polarization of the field, + for LHCP light and - for RHCP. The photon frequency ω is related to the wave vector via the dispersion relation $\omega = \frac{k}{c}$ while the λ parameter quantifies the strength of the light-matter interaction. Since $\lambda = \sqrt{\frac{\hbar}{\epsilon_0 V}}$, stronger couplings are usually obtained by extreme confinement of the quantization volume, for example in picocavities of nanoplasmonic devices.[4,5] Experiments keep moving the limit of ultrastrong coupling with the recent realizations of subnanometric cavities, i.e. $\lambda \approx 0.05$ a.u. . We point out that two photons are needed in order to consistently describe the vector potential in Eq.2. The wave function is modeled using the minimal coupling quan-

tum electrodynamics coupled cluster methodology (MC-QED-CCSD-SD)[3,6] implemented in a development version of the eT program[7]

$$|\psi\rangle = \exp\left[\mathrm{T}_1 + \mathrm{T}_2 + (\mathrm{S}_{2,\mathbf{k}} + \mathrm{S}_{1,\mathbf{k}} + \gamma_{\mathbf{k}})b^\dagger_{\mathbf{k}} + (\mathrm{S}_{2,-\mathbf{k}} + \mathrm{S}_{1,-\mathbf{k}} + \gamma_{-\mathbf{k}})b^\dagger_{-\mathbf{k}}\right] |\mathrm{HF}\rangle \otimes |0,0\rangle\,,$$

where, in addition to the usual electronic excitation operators T_1 and, T_2, electron-photon and photon excitation operators are included in the cluster operator

$$\begin{aligned}
\mathrm{T}_1 &= \sum_{ai} t^a_i E_{ai} & \mathrm{T}_2 &= \frac{1}{2}\sum_{abij} t^{ab}_{ij} E_{ai}E_{bj} \\
\mathrm{S}_{1\alpha} &= \sum_{ai} s^a_{i\alpha} E_{ai} & \mathrm{S}_{1\beta} &= \sum_{ai} s^a_{i\beta} E_{ai} \\
\mathrm{S}_{2\alpha} &= \frac{1}{2}\sum_{aibj} s^{ab}_{ij\alpha} E_{ai}E_{bj} & \mathrm{S}_{2\beta} &= \frac{1}{2}\sum_{aibj} s^{ab}_{ij\beta} E_{ai}E_{bj}.
\end{aligned} \tag{3}$$

In Eq.4, $|\mathrm{HF}\rangle$ denotes the Hartree-Fock Slater determinant and $|0,0\rangle$ is the photonic vacuum on both $b^\dagger_{\mathbf{k}}$ and $b^\dagger_{-\mathbf{k}}$ photons. The electronic operators E_{ai} excite an electron from the occupied orbitals i into the virtual orbitals a. A detailed discussion of the theoretical description of chiral cavities can be found in Ref.[3] All the calculations have been performed using a cc-pvdz basis set.[8,9] The geometries used for the reported calculations have been optimized using the Orca package with B3LYP/def2-SVP.[10] Moreover, the nudged elastic band approach[11] has been used to compute the reaction path for the benzaldehyde reaction in Fig.2. In Fig.5, the Coulomb contribution has been computed at the CCSD level subtracting the energy of the individual fragments from the energy of the two fragments at every distance.

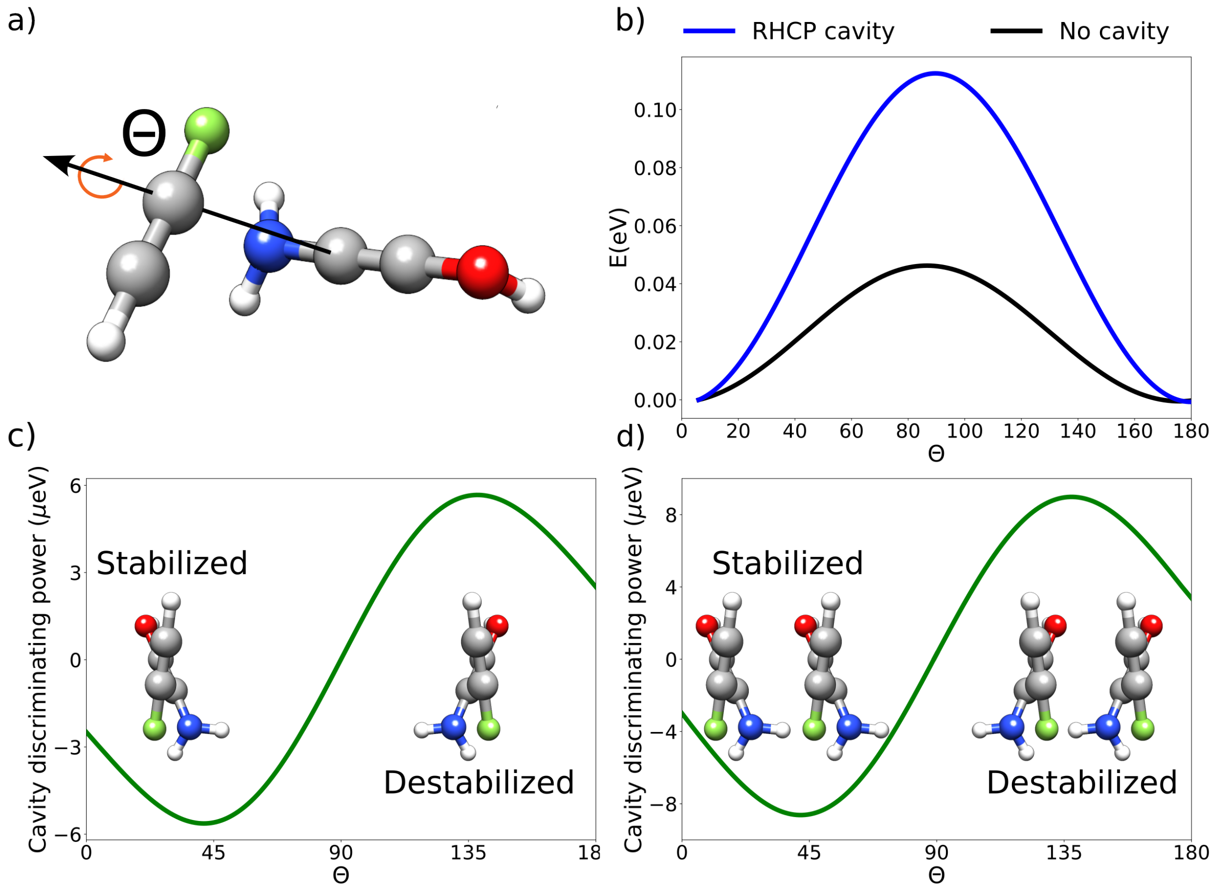

Figure 1: Cavity induced discrimination on a van der Waals chiral compound. a-b Potential energy surface for the enantiomeric interconversion inside and outside the cavity as obtained varying the angle θ. Strong coupling to the field induces a significant change in the energy profile doubling the barrier height. **c-d** Field stabilization as a function of the interconversion reaction coordinate for one or two van der Waals compounds. We note that the cavity discrimination changes sign as the complex changes its chirality and that the effect increases when the number of strongly coupled molecules is increased.

Van der Waals compounds

In most cases, enantiomers cannot be converted into each other without breaking chemical bonds. Therefore, the equilibrium composition of an enantiomeric mixture within a chiral cavity is not significantly modified due to the μeV field-induced energy differences in the ground state. However, the cavity effect should be significantly more pronounced for chiral molecules that can undergo interconversion without the need for breaking bonds. As a case study, we consider van der Waals complexes, stable systems that are held together by intermolecular forces, i.e. dispersion.[12] Specifically, the system we focus on is composed of two substituted acetylene compounds forming a cross in the most stable configuration. Rotation of either one of the two acetylene compounds along the intermolecular axis leads to enantiomeric interconversion, see Fig.1a. The energy profile of this rotation in vacuum (black) and in a LHCP cavity (blue) is shown in Fig.1b. In both cases a peak in the angular dispersion, representing the interconversion barrier, is observed at approximately $90°$. Notably, the barrier height more than doubles inside the chiral cavity increasing from 0.04 eV up to 0.1 eV. These cavity-induced effects are consistent with findings from previous literature studies.[13–15] If the chiral field contributions are isolated (subtracting for a given angle θ the energy between the molecule and its mirror image), the dispersion curve in Fig.1c is obtained. The observed trend exhibits a striking similarity to the one already discussed in Fig.3. Specifically, we recognize the sign change in the field stabilization at the $90°$, i.e. where the system's chirality is inverted. The field stabilizes the R enantiomer (θ larger than $90°$) more than the S enantiomer (θ smaller than $90°$) in a RHCP cavity. If the number of strongly coupled complexes is increased, the chirality discriminating effects also increase, as shown in Fig.1d and discussed in Ref.[3] The chiral field effects are still several orders of magnitude smaller than the energetic barrier, but their relevance in percentage is significantly larger than for normal reactive events. Moreover, the complex shown in Fig.1

is held together by dipole-dipole type interactions, i.e. the strongest intermolecular interactions that do not involve charges or hydrogen-bonds. In other van der Waals complexes held together only by dispersion forces, the field effect should be even more pronounced.

SN1 reaction and role of the electronic wave function

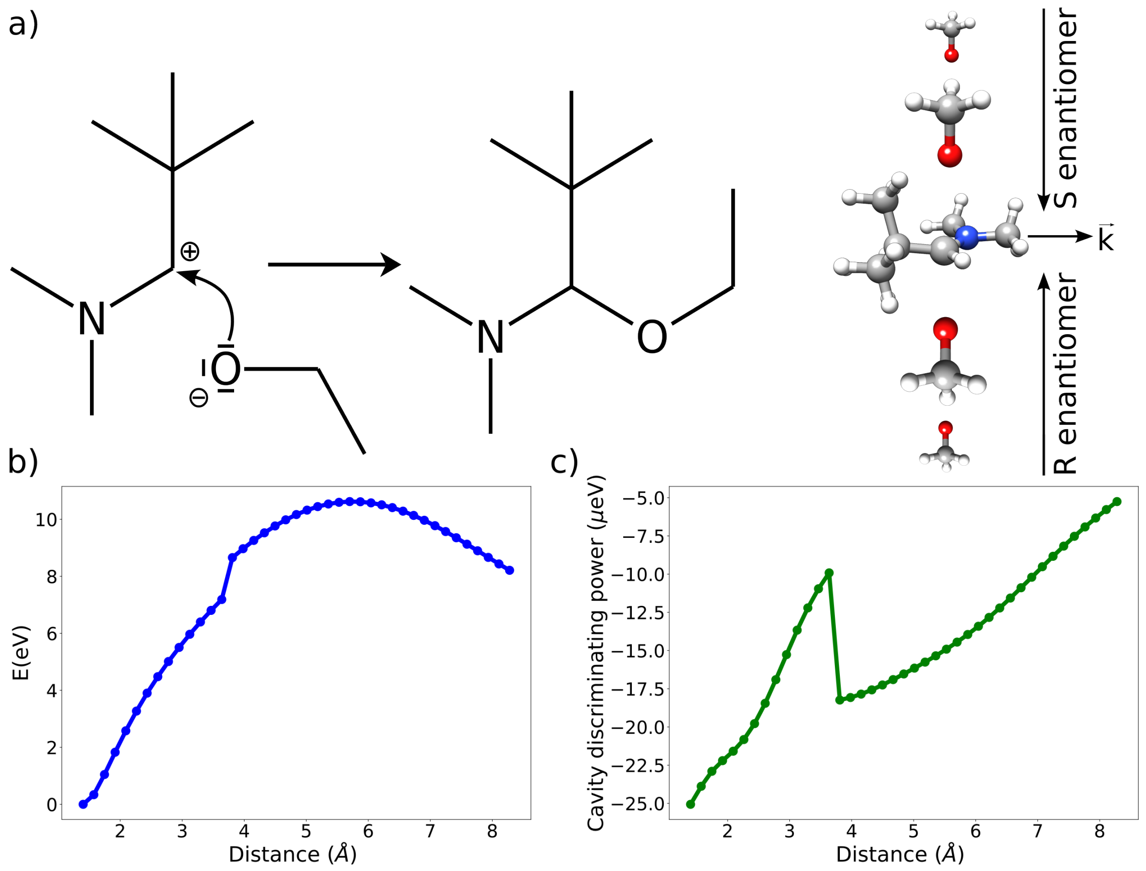

Figure 2: Field effects on a short-range SN1 reaction. **a**, Reaction mechanism and stereoselectivity for the N,N-dimethyl neopentyl amine and methanol reaction. **b**, Potential energy surface for the reaction, the methanol is approaching perpendicular to the cavity wave vector **k**. **c**, Cavity discriminating power, computed by subtracting the potential energy surfaces for top and bottom approaches, see panel **a**. We notice that the sign of the field discrimination power is the same along the full pathway.

In the main text, we discussed the field effects on the reactivity of the hemiacetal reaction.

In this section, we study the SN1 reaction between a cation of N,N-dimethyl neopentyl amine and methanol in a RHCP cavity. The cavity parameters are ω = 2.7 eV and λ = 0.05 a.u. . The reagents approach each other in the direction perpendicular to the field vector $\mathbf{k}$. Similarly to the benzaldehyde case in the main text, we notice that the cavity discriminating power has the same sign along the reactive pathway, decreasing as the distance increases. Moreover, we observe that both the potential energy surface and the field-induced discrimination exhibit a discontinuity at around 4 Å. Discontinuities in the potential energy surfaces are a well-known problem in electronic structure methods related to an abrupt change in the system wave function with the creation or breaking of chemical bonds. These discontinuities can be eliminated using a complete active space approach (CAS).[16,17] Future investigations will focus on the development of QED-CAS methodology. However, it is important to note that for the SN1 reaction in Fig.2, the discontinuities do not affect the qualitative conclusions drawn in this work. Moreover, since the reagents are closed-shell systems, there is no multireference character in the wave function at long range. Furthermore, we highlight that discontinuities in the cavity discriminating power coincide with the discontinuities in the potential energy surface. The chirality effect of the field, therefore, is sensitive to the system wave function and not only the approach geometry (which changes smoothly for the reaction in Fig.2).

Benzaldehyde reaction in the direction parallel to $\mathbf{k}$

In Fig.2 of the main paper, we display the field effects for the reaction between a benzaldehyde and an ethanol group. The approach direction in that case is chosen to be parallel to $\mathbf{k}$ with a field frequency fixed to 2.7 eV. If the reaction path is instead perpendicular to k, Fig.S3 is obtained. We notice that while both the full surface (panel b) and the cavity discrimination power (panel c) show quantitative differences compared to Fig.2 of the main text, no qualitative difference

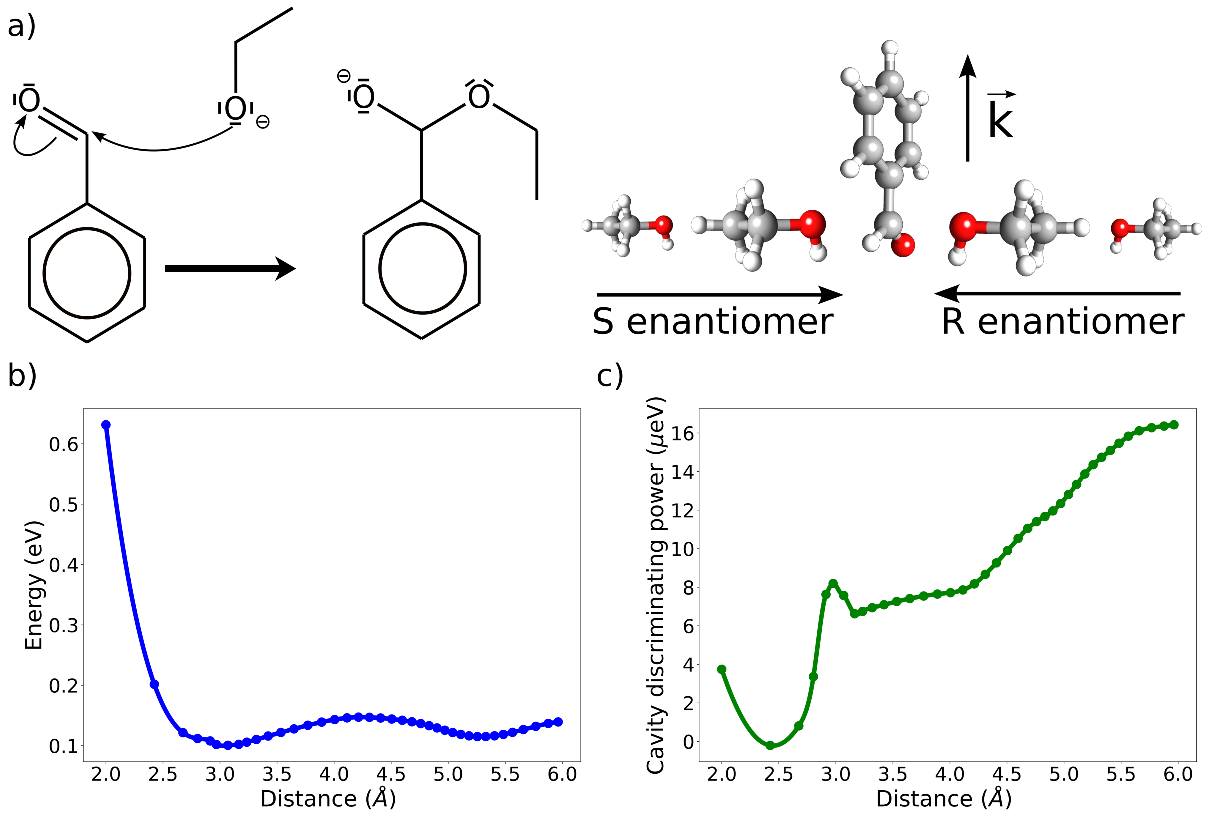

Figure 3: Short range field effects on the benzaldehyde-ethanol reaction when the groups approach each other perpendicularly to **k** **a**, Reaction mechanism for the benzaldehyde-ethanol reaction. **b**, Potential energy surface for the reaction as a function of the C-O distance. **c**, Cavity discriminating power, computed by subtracting the potential energy surfaces for S and R approaches, see panel **a**. We notice that the sign of the field discrimination power remains constant along the full pathway.

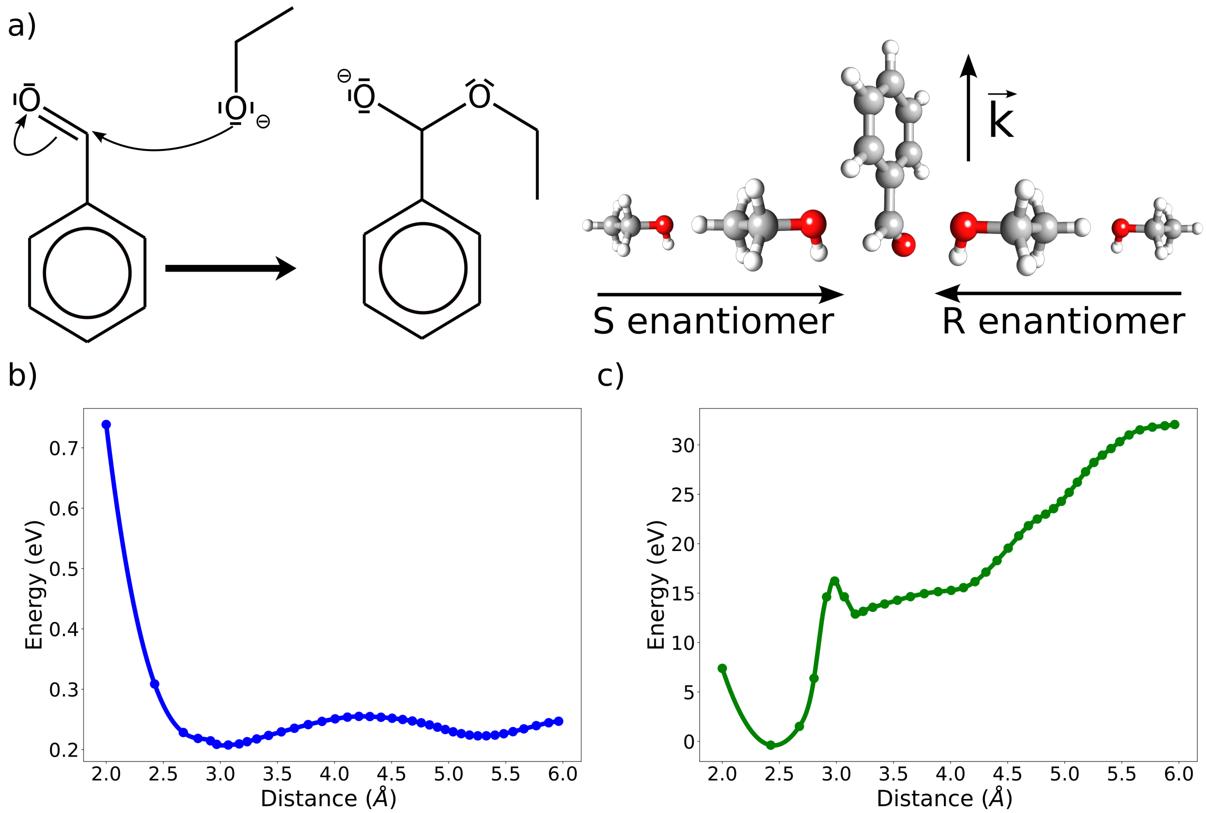

Figure 4: Short range field effects on the benzaldehyde-ethanol reaction when the groups approach each other perpendicularly to **k** **a**, Reaction mechanism for the benzaldehyde-ethanol reaction. **b**, Potential energy surface for the reaction as a function of the C-O distance. **c**, Cavity discriminating power, computed by subtracting the potential energy surfaces for S and R approaches, see panel **a**. We notice that the sign of the field discrimination power remains constant along the full pathway.

is observed as the cavity still stabilizes the R enantiomer and the sign of the stabilization is constant along the reaction coordinate. If the frequency of the cavity field is doubled from 2.7 eV to 5.4 eV, the potential energy surface does not change significantly but the field stabilization increases by almost factor two as does the optical activity of the molecule.

References

(1) Schäfer, C.; Baranov, D. G. Chiral Polaritonics: Analytical Solutions, Intuition, and Use. *J. Phys. Chem. Lett.* **2023**, *14*, 3777–3784.

(2) Baranov, D. G.; Schäfer, C.; Gorkunov, M. V. Toward Molecular Chiral Polaritons. *ACS Photonics* **2023**,

(3) Riso, R. R.; Grazioli, L.; Ronca, E.; Giovannini, T.; Koch, H. Strong coupling in chiral cavities: nonperturbative framework for enantiomer discrimination. *Phys. Rev. X.* **2023**, *13*, 031002.

(4) Chikkaraddy, R.; De Nijs, B.; Benz, F.; Barrow, S. J.; Scherman, O. A.; Rosta, E.; Demetriadou, A.; Fox, P.; Hess, O.; Baumberg, J. J. Single-molecule strong coupling at room temperature in plasmonic nanocavities. *Nature* **2016**, *535*, 127–130.

(5) Santhosh, K.; Bitton, O.; Chuntonov, L.; Haran, G. Vacuum Rabi splitting in a plasmonic cavity at the single quantum emitter limit. *Nat. Commun.* **2016**, *7*, ncomms11823.

(6) Haugland, T. S.; Ronca, E.; Kjønstad, E. F.; Rubio, A.; Koch, H. Coupled cluster theory for molecular polaritons: Changing ground and excited states. *Phys. Rev. X* **2020**, *10*, 041043.

(7) Folkestad, S. D.; Kjønstad, E. F.; Myhre, R. H.; Andersen, J. H.; Balbi, A.; Coriani, S.; Giovannini, T.; Goletto, L.; Haugland, T. S.; Hutcheson, A. et al. eT 1.0: An open source electronic structure program with emphasis on coupled cluster and multilevel methods. *J. Chem. Phys.* **2020**, *152*.

(8) Pritchard, B. P.; Altarawy, D.; Didier, B.; Gibsom, T. D.; Windus, T. L. A New Basis Set Exchange: An Open, Up-to-date Resource for the Molecular Sciences Community. *J. Chem. Inf. Model.* **2019**, *59*, 4814–4820.

(9) Dunning, T. H. Gaussian basis sets for use in correlated molecular calculations. I. The atoms boron through neon and hydrogen. *J. Chem. Phys.* **1989**, *90*, 1007–1023.

(10) Neese, F. Software update: The ORCA program system—Version 5.0. *Wiley Interdiscip. Rev. Comput. Mol. Sci.* **2022**, *12*, e1606.

(11) Henkelman, G.; Uberuaga, B. P.; Jónsson, H. A climbing image nudged elastic band method for finding saddle points and minimum energy paths. *J. Chem. Phys.* **2000**, *113*, 9901–9904.

(12) Sutter, P.; Wimer, S.; Sutter, E. Chiral twisted van der Waals nanowires. *Nature* **2019**, *570*, 354–357.

(13) Pavošević, F.; Smith, R. L.; Rubio, A. Computational study on the catalytic control of endo/exo Diels-Alder reactions by cavity quantum vacuum fluctuations. *Nat. Commun.* **2023**, *14*, 2766.

(14) Riso, R. R.; Haugland, T. S.; Ronca, E.; Koch, H. Molecular orbital theory in cavity QED environments. *Nat. Commun.* **2022**, *13*, 1368.

(15) Pavosevic, F.; Smith, R. L.; Rubio, A. Cavity Click Chemistry: Cavity-Catalyzed Azide–Alkyne Cycloaddition. *J. Phys. Chem. A* **2023**, *127*, 10184–10188.

(16) Nottoli, T.; Gauss, J.; Lipparini, F. Second-order CASSCF algorithm with the Cholesky decomposition of the two-electron integrals. *J. Chem. Theory Comput.* **2021**, *17*, 6819–6831.

(17) Roos, B. O.; Taylor, P. R.; Sigbahn, P. E. A complete active space SCF method (CASSCF) using a density matrix formulated super-CI approach. *Chem. Phys.* **1980**, *48*, 157–173.
